# A simple mathematical model of allometric exponential growth describes the early three-dimensional growth dynamics of secondary xylem in Arabidopsis roots

**DOI:** 10.1098/rsos.190126

**Published:** 2019-03-06

**Authors:** Anna Thamm, Sabina Sanegre-Sans, Jennifer Paisley, Susana Meader, Ana Milhinhos, Sonia Contera, Javier Agusti

**Affiliations:** 1 Plant Sciences Department, University of Oxford, South Parks Road, Oxford OX1 3RB, UK; 2 Sir William Dunn School of Pathology, University of Oxford, South Parks Road, Oxford OX1 3RE, UK; 3 Clarendon Laboratory, Physics Department, University of Oxford, Parks Road, Oxford OX1 3PU, UK

**Keywords:** allometric growth, Arabidopsis, model, development, xylem

## Abstract

Unravelling the specific growth dynamics of key tissues and organs is fundamental to understand how multicellular organisms orchestrate their different growth programmes. In plants, the secondary growth (thickening) of stems and roots provides the mechanical support that plants need to achieve their developmental potential. We used conventional anatomical and microscopy techniques, image-processing software, and quantitative analysis to understand and mathematically describe the growth dynamics of the early developmental stages of secondary xylem (the main tissue developed during secondary growth). Results show that such early developmental stages are characterized by exponential expansion of secondary xylem in three dimensions in the form of an inverted cone, with a power law that describes the relationship between the area of the base and the longitudinal progression (height) of the growing secondary xylem cone over time with a scaling exponent of 2/5: the signature of allometric growth. Our work constitutes a starting point for future modelling of secondary xylem in particular and secondary growth in general.

## 1. Introduction

In plants, secondary growth is the process by which stems and roots grow in girth. This is a pivotal process in development that provides plants with the mechanical support and stability that they need to expand their growth capacities [1]. Secondary growth is the result of the formation of secondary vascular tissues: secondary xylem and secondary phloem. Such secondary vascular tissues emerge from the activity of a highly specialized pool of stem cells (meristem) termed cambium [2]. The cambium develops concentric cylinders of secondary phloem centrifugally and secondary xylem centripetally [3]. The concentric accumulation of such tissues leads to secondary growth and thus thickening of stems and roots. Secondary xylem proliferates and contributes more to the mechanical support of plants than secondary phloem. Together with mechanical support, the fundamental role of xylem is the acropetal transport of water and solutes from the soil to all plant cells [2]. In trees, secondary xylem brings about wood: one of the largest sources of terrestrial biomass, the largest sink of atmospheric CO_2_ after oceans and a main source of raw material for the renewable energy, construction or timber industries [4].

Secondary xylem can be found not only in woody but also in many herbaceous species, such as the model system *Arabidopsis thaliana*, which has proven to be an excellent model system to study secondary growth [5].

Despite the pivotal importance of secondary growth for plant development and industry, our knowledge about the biology of the process is still rather fragmented. Work within the last decades has revealed new aspects about the hormonal and genetic control of secondary growth (for review see Miyashima *et al*. [6]). A major further step forward towards our understanding of the process would be to establish equations that could allow for the generation of predictive models. With such view in mind and to take a first step in that direction, we focused on understanding the growth dynamics of the early stages of secondary xylem development in Arabidopsis roots. Using conventional anatomical and microscopy techniques coupled with automated image quantification, we defined equations explaining such early stages of secondary xylem development. Geometrically, in roots, secondary xylem development occurs in the form of an inverted cone which volume expands exponentially with time [7]. Our observations indicate that the quantitative data of the cone growth fit a simple mathematical model of allometric exponential growth, implying high coordination between the expansion area and the longitudinal progression during the early stages of secondary xylem development. We suggest that (i) our work can be used as a starting point for modelling secondary xylem development and (ii) in the future, a model for secondary growth could be developed by generating data for the other tissues contributing to secondary growth (e.g. phloem and cambium) and by integrating genetic, environmental and physiological data.

## 2. Methods and materials

### 2.1. Plant materials and growth conditions

The *Arabidopsis thaliana* Col-0 ecotype was used. In all cases, seeds were sterilized, stratified and grown vertically in 12 × 12 cm plates containing MS media (21°C constant temperature and 16 h light/8 h dark regime) as described in [8].

### 2.2. Sampling, sectioning and imaging

Seedlings for developmental stages analyses grew for 5, 6, 7, 11, 15 or 21 days after germination (from here on DAG). The base (uppermost part) of the root was harvested, embedded in 1% agarose and fixed in 4% paraformaldehyde (PFA; Sigma) overnight. Agarose blocks containing the root were then dehydrated and embedded in Technovit 7100 (Heraeus Kulzer, EBSciences) as described in [9]. Sections (7–10 µm) were generated using a Microm HM335E microtome. Samples used for secondary xylem cell count, area measurement and longitudinal progression of secondary growth analyses were harvested at 7, 11, 15 or 21 DAG (as described above), infiltrated and wax-embedded as described in [4]. Sections (5 µm) were generated using a Leica RM2135 microtome. In all cases, sections were mounted on water on glass slides and stained with Toluidine Blue (0.05%) as described in [4]. Images were acquired using a brightfield Olympus BX50 light microscope equipped with a q Imaging RETIGA Exi camera and the Image Pro Plus software.

### 2.3. Quantitative analyses of secondary xylem

**Figure 1.**
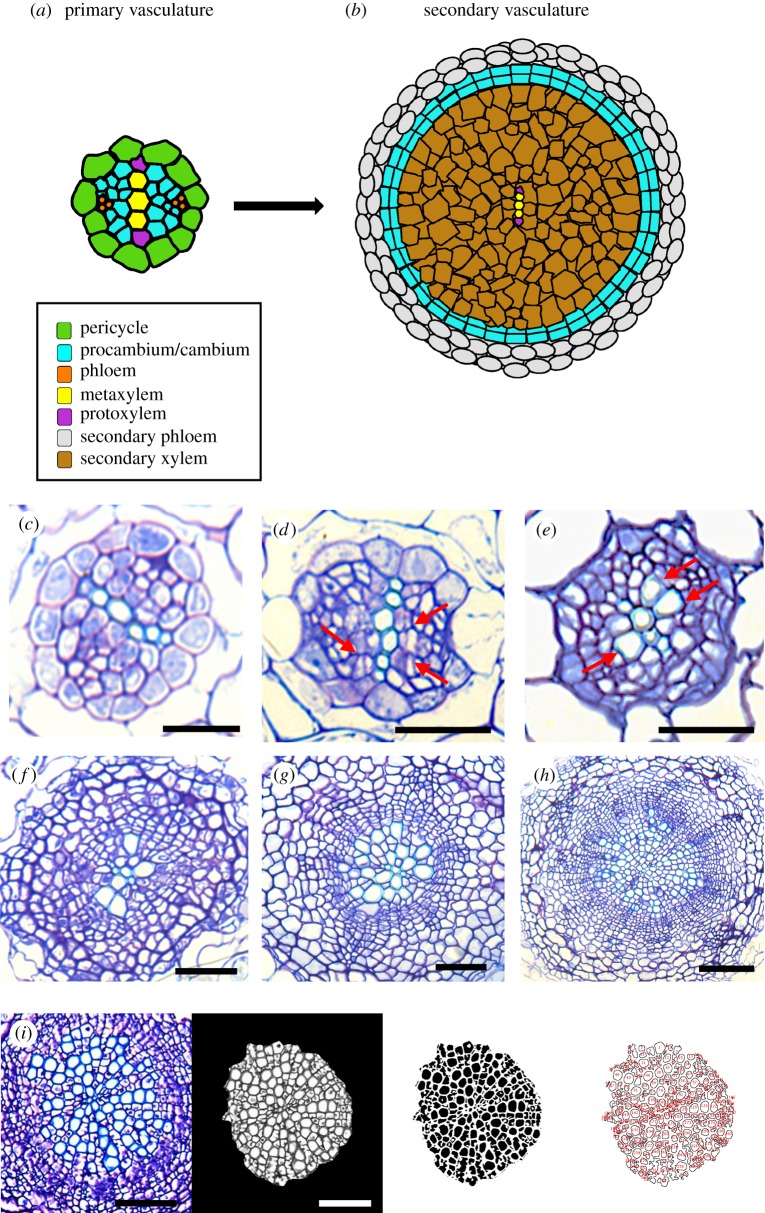
Secondary xylem development in Arabidopsis roots. (*a*,*b*) Primary to secondary development transition. (*c*–*h*) Transversal sections at the base of the root of *Arabidopsis thaliana* representing key developmental stages of secondary growth. (*c*) 5 DAG, (*d*) 6 DAG; arrows indicate procambial cells dividing, (*e*) 7 DAG; arrows indicate newly developed secondary xylem cells, (*f*) 11 DAG, (*g*) 15 DAG, (*h*) 21 DAG. Scale bars: *c*, *d*, *e*: 25 µm; *f*, *g*: 50 µm; *h*: 100 µm. (*i*) Example of secondary xylem cell counting using our customized macro for ImageJ. Scale bars: 50 µm.

Image analyses and automated quantification were performed using ImageJ software [10]. We developed an automated method for cell count quantification (electronic supplementary material, document S1). Manual cell count revealed that our method displayed an accuracy of at least 95%. Secondary xylem area was measured using standard ImageJ tools. The longitudinal progression of secondary xylem development along the root was determined through successive histological sections on wax-embedded samples that represented the entire root. For each analysed developmental time point (7, 11, 15 and 21 DAG), successive 0.5 cm samples were collected along the root, starting from the base towards the root apical meristem. Each 0.5 cm sample was wax-embedded, sectioned (5 µm), stained and photographed as described above, ensuring that the entire root could be analysed and that our precision was at the range of ±5 µm. For each root, the longitudinal extension of secondary xylem proliferation was assessed as the last position (5 µm section) within the root where we could observe secondary vascular cells (i.e. in the next section only primary xylem was detected; transversal sections of roots looking like figure 1*a*, left panel or *c*). In all cases, experiments were repeated three times (details provided in electronic supplementary material, Thammetal_2019_SupplTable1).

The cone volume (*V*) was calculated using the xylem area and longitudinal extension at 7, 11, 15 and 21 DAG. The number of cells and the volume of the conical shape was plotted versus time and fitted to exponential growth models $V=V_{o}e^{kt}$ and $N=N_{o}e^{k_{2}t}$ for the volume of the cylinder and the number of cells respectively using an orthogonal distance regression nonlinear fit method implemented with the Origin Pro 9.1 software. Three-dimensional graphics were plotted with the software Wolfram Research Mathematica 9.0. For the power law fit we used the Levenberg–Marquardt algorithm.

## 3. Results

### 3.1. Dynamics of early developmental stages of secondary xylem

We performed anatomical analyses on the growth dynamics of the early stages of secondary xylem to identify key developmental stages on which to collect our quantitative data for subsequent mathematical analyses. Figure 1 shows a time-lapse of the most representative stages of secondary xylem development. In our growing conditions, at 5 days after germination (DAG), we could clearly observe the typical diarch organization of the primary vasculature (figure 1*c*). Anticlinal and periclinal cell divisions were first observed within the procambium zone at 6 DAG (figure 1*d*). This was more evident at 7 DAG, when, in addition, the first secondary xylem cells were differentiated from the newly divided procambial cells (figure 1*e*). The time period from 7 to 11 DAG was strongly marked by cambial cellular proliferation. At 11 DAG the vascular cambium already exhibited a clear ring-like organization surrounding the secondary xylem cells that were already present (figure 1*f*). From 11 DAG to 21 DAG secondary xylem cells proliferated in a high rate (see figure 1*f*,*g*).

Taken all together, secondary xylem starts differentiating at 7 DAG and expands in proliferation between 7 and 21 DAG.

### 3.2. Allometric three-dimensional secondary xylem expansion

**Figure 2.**
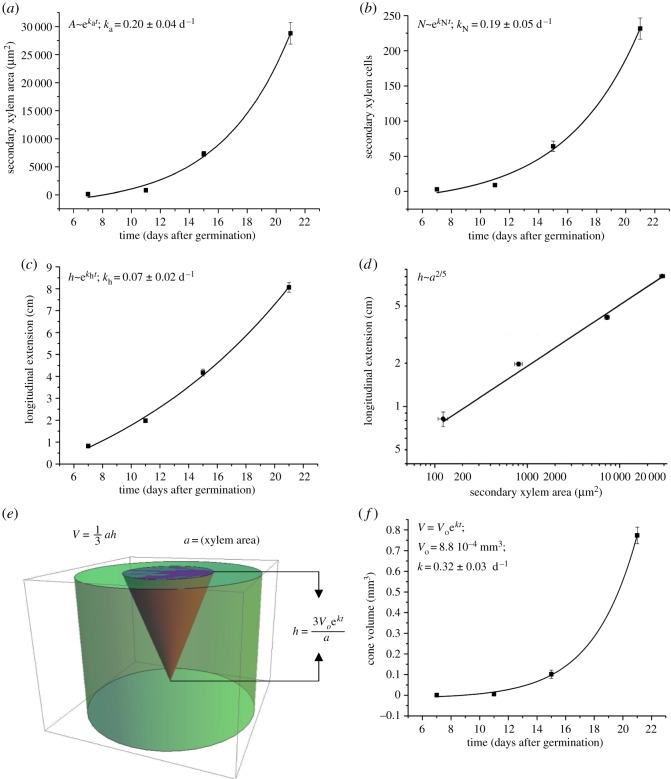
Secondary xylem quantitative analyses and fittings (*a*–*d*). (*a*) Secondary xylem area progression at the base of the root. Black squares: secondary xylem area (µm^2^). Black curve: exponential growth fit. (*b*) Amount of secondary xylem cells at the base of the root. Black squares: number of secondary xylem cells. Black curve: exponential growth fit. (*c*) Basipetal (longitudinal) progression of secondary xylem along the root. Black squares: longitudinal extension (cm). Black curve: exponential growth fit. (*d*) Log-log graphical representation plotting the secondary xylem area in the *x* axis and the basipetal extension of secondary growth in the *y* axis. Black squares: values for 7, 11, 15 or 21 DAG. (*e*) Three-dimensional graphical representation of secondary xylem growth over time in the root of Arabidopsis (*V* = *V*_0_ e*^kt^*). (*f*) Exponential growth of the cone in two dimensions over time: fit to exponential growth. Error bars are propagated errors in all cases.

Surface (area) expansion measurements at the base (uppermost part) of the root at 7, 11, 15 and 21 DAG revealed an average area of 1519.56 µm^2^ at 7 DAG, 9881.08 µm^2^ at 11 DAG, 89373.70 µm^2^ at 15 DAG and 352557.62 µm^2^ at 21 DAG (figure 2*a*). Using our ImageJ-based automated quantification method (macro) for secondary xylem cell counting on the basal part of the roots (electronic supplementary material, SupplDoc1), we detected an average of 3 secondary xylem cells at 7 DAG, 8.92 at 11 DAG, 64.19 at 15 DAG and 231.57 at 21 DAG (figure 2*b*; for cell counting method see figure 1*i*). Xylem area and cellular proliferation exponential expansion (figure 2*a*,*b*; electronic supplementary material, SupplDoc2) occurs in a such similar manner that the exponential constant (*k*) was almost identical in both cases, being *k*_a_ (constant for the area expansion, in days^−1^ (d^−1^)) = 0.20 ± 0.04 d^−1^ and *k_N_* (constant for the cellular proliferation) = 0.19 ± 0.05 d^−1^ (figure 2*a*,*b*; electronic supplementary material, file S2). Therefore, we decided to work exclusively with geometrical approximations (surface) rather than with cellular proliferation for further mathematical analyses.

The progression of the longitudinal extension of secondary xylem on the studied time frame was also exponential (figure 2*c*). Indeed, at 7 DAG the secondary xylem extended for 0.2805 cm, progressing to 1.975 cm at 11 DAG, 4.175 cm at 15 DAG and 8.06 cm at 21 DAG. Remarkably, although the radial expansion of secondary xylem occurred in the range of µm^2^ and the longitudinal extension is found in the range of cm, Pearson correlation test revealed high correlation (0.97486) between the two types of growth over time (at 7, 11, 15 and 21 DAG), indicating that tight correlation between them is necessary. When plotting the area of the radial growth against the longitudinal growth, the data fitted almost perfectly (*R*^2^ is 0.9932) a power law with an exponent of 2/5, which is the signature of allometric growth (within the time range studied here) (figure 2*d*; electronic supplementary material, notes S2). All measurements are contained in electronic supplementary material, Thammetal2019_2019_SupplTable1.

### 3.3. A simple growth model that explains secondary xylem growth dynamics in three dimensions

We approximated the secondary xylem volume within the roots (as a whole three-dimensional tissue) by a cone (figure 2*e*). We defined the secondary xylem area *a* at the base of the root as the base of the cone, and the longitudinal extension of secondary xylem as height *h*. Using our quantitative data for *a* and *h* at 7, 11, 15 and 21 DAG we first calculated the volume *V* of the conical shape that *a* and *h* form at each specific time point and, then, we plotted the evolution of *V* over time (*t*) (figure 2*f*). Our analyses revealed that the expansion of the conical volume over time clearly fits again an exponential growth behaviour $V=V_{o}e^{kt}$, in which our growth constant (*k*) equals to 0.32 d^−1^ and the initial volume (*V*_0_) is 8.8 × 10^−4^ mm^3^. In this way, the volume of the cone is a function of the initial volume, and it progresses exponentially with time. The fit converges very well and, indeed, our coefficient of determination *R*^2^ is 0.9999996.

The exponential fit implies that at this stage of growth the volume change satisfies a linear differential equation, i.e. dV/dt=k V, which corresponds to a particular, simplified case of the Lockhart equation [11]. Considering the cone volume ($V=(1/3)\;\pi r^{2}h$), the evolution of the longitudinal extension (*h*) of xylem along the root over time (*t*) can then be inferred:

$$h=\frac{3V_{o}e^{kt}}{a}=\frac{3V_{o}e^{kt}}{\pi r^{2}},$$

where *r* is the radius of the cone. Taken all together, our results fit a simple exponential model that describes the growth dynamics of the early stages of secondary xylem development in Arabidopsis roots in three dimensions over time with an allometric exponent of 2/5.

## 4. Discussion

Determining the growth dynamics of key tissues in development is paramount to understand how organisms develop, because it provides a broad view of how the different growth programmes interact with each other and regulate each other during development. In plants, secondary growth occurs mainly in stems and roots. While modelling in stems is scarce, modelling in roots emerged in the 1970s and has provided very valuable information [12]. However, even though some of those models took into account thickening, little attention has been paid to secondary vascular tissue development as a process and reports on mathematical models of developmental kinetics and/or the growth dynamics of secondary growth/secondary vascular tissues are almost non-existent. A recent (and pioneer) work elegantly analysed the morphodynamics progression of tissue type ratios of xylem and phloem on two dimensions over time [13]. Here, we present a simple model describing the early stages of secondary xylem growth dynamics in three dimensions. We show that, during such early stages, secondary xylem three-dimensional growth follows an allometric-function model.

This is a first step towards modelling secondary xylem development in particular and, in general, secondary growth as a process. It is expected that, by generating new experimental data, it will be possible to expand our equation to later xylem developmental stages and to incorporate in it the effect that genetics and the environment exert on the control of the process. Furthermore, by extending our experimentation to secondary phloem development, phellogen formation, phelloderm development and the transition from procambium to vascular cambium rearrangement (all of them fundamental processes that take place during secondary growth [2]) it may be possible to generate a full model for secondary growth.

Considering that the conical shape that the secondary xylem displays (in three dimensions) in Arabidopsis roots resembles that of the stem of some of the most relevant trees for industry, we suggest that our approach may be exportable to trees as a valuable tool to (i) understand new aspects of tree physiology and development and (ii) expand our capabilities for wood yield prediction.

Taken all together, our study provides a mathematical description of the growth dynamics of the early stages of secondary xylem development. Our work constitutes a starting point to perform new experimentation aimed at mathematically modelling secondary xylem development and, in the long term, secondary growth as a process. We suggest that our data and our experimentation procedures can be transferred to trees to assist both basic and applied scientific experimentation.

## Supplementary Material

ImageJ macro to count secondary xylem cells

Reviewer comments

## Supplementary Material

Fit to Exponential

## Supplementary Material

Supplementary Table 1_Cell count, areas and longitudinal extension

## Data accessibility

All data used for this study are described in electronic supplementary material, table S1.

## References

[1] Spicer R, Groover A 2010 Evolution of development of vascular cambia and secondary growth. New Phytol. 186, 577–592. (10.1111/j.1469-8137.2010.03236.x) 20522166

[2] Esau K 1961 Anatomy of seed plants. John Wiley & Sons, Inc.

[3] Suer S, Agusti J, Sanchez J, Schwarz M, Greb T 2011 *WOX4* imparts auxin responsiveness to cambium cells in *Arabidopsis*. Plant Cell 23, 3247–3259. (10.1105/tpc.111.087874) 21926336 PMC3203433

[4] Agusti J, Lichtenberger R, Schwarz M, Nehlin L, Greb T 2011 Characterization of transcriptome remodeling during cambium formation identifies *MOL1* and *RUL1* as opposing regulators of secondary growth. PLoS Genet. 7, e1001312 (10.1371/journal.pgen.1001312) 21379334 PMC3040665

[5] Chaffey N, Cholewa E, Regan S, Sundberg B 2002 Secondary xylem development in *Arabidopsis*: a model for wood formation. Physiol. Plant. 114, 594–600. (10.1034/j.1399-3054.2002.1140413.x) 11975734

[6] Miyashima S, Sebastian J, Lee J-Y, Helariutta Y 2012 Stem cell function during plant vascular development. EMBO J 32, 178–193. (10.1038/emboj.2012.301) 23169537 PMC3553377

[7] Savidge RA 1993 Formation of annual rings in trees. In Oscillations and morphogenesis (ed. Rensing L), pp. 343–363. New York, NY: Marcel Dekker.

[8] Meijon M, Satbhai SB, Tsuchimatsu T, Busch W 2014 Genome-wide association study using cellular traits identifies a new regulator of root development in *Arabidopsis*. Nat. Genet. 46, 77–81. (10.1038/ng.2824) 24212884

[9] Proust H, Honkanen S, Jones VAS, Morieri G, Prescott H, Kelly S, Ishizaki K, Kphchi T, Dolan L 2016 RSL class I genes controlled the development of epidermal structures in the common ancestor of land plants. Curr. Biol. 26, 93–99. (10.1016/j.cub.2015.11.042) 26725198 PMC4712171

[10] Abramoff MD. MPJ, Ram SJ 2004 Image processing with ImageJ. Biophotonics International 11, 36–42.

[11] Lockhart JA 1965 An analysis of irreversible plant cell elongation. J. Theor. Biol. 8, 264–266. (10.1016/0022-5193(65)90077-9) 5876240

[12] Dupuy L, Gregory PJ, Bengough AG 2010 Root growth models: towards a new generation of continuous approaches. J. Exp. Bot. 61, 2131–2143. (10.1093/jxb/erp389) 20106912

[13] Sankar M, Nieminen K, Ragni L, Xenarios I, Hardtke CS 2014 Automated quantitative histology reveals vascular morphodynamics during Arabidopsis hypocotyl secondary growth. Elife 3, e01567 (10.7554/eLife.01567) 24520159 PMC3917233

