# Supplementary material for: A simple mathematical model of allometric exponential growth describes the early three-dimensional growth dynamics of secondary xylem in Arabidopsis roots
Source: R Soc Open Sci. 2019 Mar 6;6(3):190126. doi: 10.1098/rsos.190126 (PMC6458390; doi:10.1098/rsos.190126)
Supplement: ImageJ macro to count secondary xylem cells [file rsos190126supp1.docx]

**ImageJ macro to count secondary xylem cells**

setAutoThreshold("Default dark");

run("Threshold...");

setThreshold(158, 255);

run("Make Binary", "thresholded remaining black");

run("Analyze Particles...", "size=16-Infinity circularity=0.00-1.00 show=Outlines display exclude summarize");
