# Supplementary material for: A simple mathematical model of allometric exponential growth describes the early three-dimensional growth dynamics of secondary xylem in Arabidopsis roots
Source: R Soc Open Sci. 2019 Mar 6;6(3):190126. doi: 10.1098/rsos.190126 (PMC6458390; doi:10.1098/rsos.190126)
Supplement: Fit to Exponential [file rsos190126supp2.docx]

**Fit to Exponential**

**Secondary xylem cell count**

| Model | ExpGro1 |  |  |
| --- | --- | --- | --- |
| Equation | y = A1*exp(x/t1) + y0 | | |
| Reduced Chi-Sqr | 118.0576 | | |
|  |  | Value | Standard Error |
| Cell count | y0 | -18.4606 | 20.01629 |
| Cell count | A1 | 4.28778 | 4.43523 |
| Cell count | t1 | 5.16293 | 1.22879 |
| Cell count | k | 0.19369 | 0.0461 |
| Cell count | tau | 3.57867 | 0.85173 |

**Secondary xylem area**

| Model | ExpGro1 |  |  |
| --- | --- | --- | --- |
| Equation | y = A1*exp(x/t1) + y0 | | |
| Reduced Chi-Sqr | 1.47553E6 | | |
|  |  | Value | Standard Error |
| Area | y0 | -2154.40545 | 2059.66403 |
| Area | A1 | 407.13176 | 392.36807 |
| Area | t1 | 4.84782 | 1.01768 |
| Area | k | 0.20628 | 0.0433 |
| Area | tau | 3.36026 | 0.7054 |

**Basipetal progression (longitudinal extension)**

| Model | ExpGro1 |  |  |
| --- | --- | --- | --- |
| Equation | y = A1*exp(x/t1) + y0 | | |
| Reduced Chi-Sqr | 0.05839 | | |
|  |  | Value | Standard Error |
| Longitudinal Ext | y0 | -3.62739 | 2.40277 |
| Longitudinal Ext | A1 | 2.66755 | 1.79168 |
| Longitudinal Ext | t1 | 14.19663 | 4.55411 |
| Longitudinal Ext | k | 0.07044 | 0.0226 |
| Longitudinal Ext | tau | 9.84035 | 3.15667 |
