# Supplementary material for: A simple mathematical model of allometric exponential growth describes the early three-dimensional growth dynamics of secondary xylem in Arabidopsis roots
Source: R Soc Open Sci. 2019 Mar 6;6(3):190126. doi: 10.1098/rsos.190126 (PMC6458390; doi:10.1098/rsos.190126)
Supplement: Reviewer comments [file rsos190126_review_history.pdf]

THE ROYAL SOCIETY
PUBLISHING

ROYAL SOCIETY
OPEN SCIENCE

A simple mathematical model of allometric exponential growth describes the early three-dimensional growth dynamics of secondary xylem in Arabidopsis roots

Anna Thamm, Sabina Sanegre-Sans, Jennifer Paisley, Susana Meader, Ana Milhinhos, Sonia Contera and Javier Agusti

Article citation details
R. Soc. open sci. **6**: 190126.
http://dx.doi.org/10.1098/rsos.190126

Review timeline
Original submission: 23 January 2019
Final acceptance: 24 January 2019

Note: Reports are unedited and appear as submitted by the referee. The review history appears in chronological order.

Note: This manuscript was transferred from another Royal Society journal with peer review.

Review History

RSOS-190126.R0 (Original submission)

Decision letter (RSOS-190126.R0)

24-Jan-2019

Dear Dr Agusti,

I am pleased to inform you that your manuscript entitled "A simple mathematical model of allometric exponential growth describes the early 3D growth dynamics of secondary xylem in Arabidopsis roots." is now accepted for publication in Royal Society Open Science.

You can expect to receive a proof of your article in the near future. Please contact the editorial office (openscience_proofs@royalsociety.org and openscience@royalsociety.org) to let us know if you are likely to be away from e-mail contact. Due to rapid publication and an extremely tight schedule, if comments are not received, your paper may experience a delay in publication.

Reports © 2019 The Reviewers; Decision Letters © 2019 The Reviewers and Editors; Responses © 2019 The Reviewers, Editors and Authors. Published by the Royal Society under the terms of the Creative Commons Attribution License http://creativecommons.org/licenses/by/4.0/, which permits unrestricted use, provided the original author and source are credited

Royal Society Open Science operates under a continuous publication model (http://bit.ly/cpFAQ). Your article will be published straight into the next open issue and this will be the final version of the paper. As such, it can be cited immediately by other researchers. As the issue version of your paper will be the only version to be published I would advise you to check your proofs thoroughly as changes cannot be made once the paper is published.

On behalf of the Editors of Royal Society Open Science, we look forward to your continued contributions to the Journal.

Kind regards,
Royal Society Open Science Editorial Office
Royal Society Open Science
openscience@royalsociety.org

on behalf of Professor Pietro Cicuta (Subject Editor)
openscience@royalsociety.org

Follow Royal Society Publishing on Twitter: @RSocPublishing
Follow Royal Society Publishing on Facebook:
https://www.facebook.com/RoyalSocietyPublishing.FanPage/
Read Royal Society Publishing's blog: https://blogs.royalsociety.org/publishing/
